# Edge mixing dynamics in graphene *p*–*n* junctions in the quantum Hall regime

**DOI:** 10.1038/ncomms9066

**Published:** 2015-09-04

**Authors:** Sadashige Matsuo, Shunpei Takeshita, Takahiro Tanaka, Shu Nakaharai, Kazuhito Tsukagoshi, Takahiro Moriyama, Teruo Ono, Kensuke Kobayashi

**Affiliations:** 1 Department of Physics, Osaka University, Toyonaka, Osaka 560-0043, Japan; 2 Institute for Chemical Research, Kyoto University, Uji, Kyoto 611-0011, Japan; 3 WPI-MANA, NIMS, Tsukuba, Ibaraki 305-0044, Japan

## Abstract

Massless Dirac electron systems such as graphene exhibit a distinct half-integer quantum Hall effect, and in the bipolar transport regime co-propagating edge states along the *p*–*n* junction are realized. Additionally, these edge states are uniformly mixed at the junction, which makes it a unique structure to partition electrons in these edge states. Although many experimental works have addressed this issue, the microscopic dynamics of electron partition in this peculiar structure remains unclear. Here we performed shot-noise measurements on the junction in the quantum Hall regime as well as at zero magnetic field. We found that, in sharp contrast with the zero-field case, the shot noise in the quantum Hall regime is finite in the bipolar regime, but is strongly suppressed in the unipolar regime. Our observation is consistent with the theoretical prediction and gives microscopic evidence that the edge states are uniquely mixed along the *p*–*n* junction.

## Abstract (web summary)

A graphene *p*–*n* junction can be created by connecting electrical gates that generate electron-doped and hole-doped areas in a flake. Here, the authors use shot-noise measurements to provide microscopic evidence that edge states are uniquely mixed along the junction in the quantum Hall regime.

## Graphical abstract

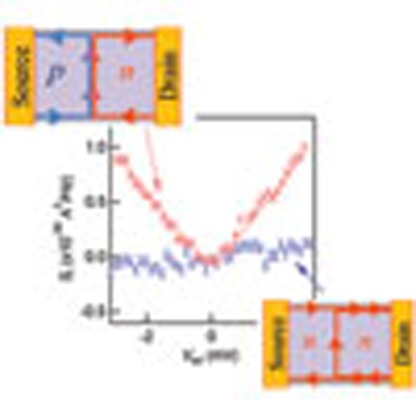

Quantum Hall (QH) edge states are an ideal platform to investigate how chirality manifests itself in electron transport. They have been extensively studied in two-dimensional electron gases (2DEG) in GaAs/AlGaAs heterostructures, where electronic beam splitters^1^,^2^ and interferometers^3^,^4^ were demonstrated by using circulating edge states. In addition to the conventional conductance measurement, the shot noise was measured in these studies to microscopically address the edge transport, as it provide information on how electrons are transmitted or reflected at potential barriers; for example, those formed by quantum point contacts. By virtue of the sensitivity of the shot noise to this partitioning process, researchers have clarified several fundamental facts about the edge states, such as the fermionic properties of electrons^1^ and the unique elementary excitation in the fractional QH regime^5^,^6^. However, as shown in Fig. 1a, only a specific chirality can be realized in a conventional 2DEG at a given direction of the magnetic field.

Graphene^7^,^8^ is now providing new insight into edge transport; reflecting the massless Dirac electrons and the resulting half-integer QH state, both edge chiralities can coexist in a single graphene device when *p* and *n* regions are formed there (namely, the ‘bipolar' regime). In reality, the lateral *p*–*n* junctions shown in Fig. 1b are realized in locally gated graphene, and the unique QH edge transport was clarified experimentally^9^ and theoretically^10^. It was found in the bipolar regime that the QH edge transport at the junction shows a peculiar fractional quantized conductance in the units of conductance quantum *e*^2^/*h*, where *h* is Planck's constant^9^. This exotic observation was phenomenologically attributed to the peculiar electron partition caused by uniform mixing of the QH edge states that co-propagate along the *p*–*n* junction^9^,^10^, which has been supported by intensive experimental works^11^,^12^,^13^,^14^,^15^,^16^,^17^. However, shot-noise measurements could directly provide a deeper insight into the microscopic dynamics of the electron partition process on the *p*–*n* junction. Theory predicted that the noise is expected to be finite in the bipolar regime, whereas it should disappear in the unipolar regime^10^. Experiments in this direction, however, have been lacking so far.

Here we present noise measurements in graphene *p*–*n* junctions in the QH regime and at zero magnetic field. The shot noise in the QH regime is finite in the bipolar regime, but is strongly suppressed in the unipolar regime, contrasting sharply with the zero-field case. The deduced Fano factors are consistent with the theoretical prediction^10^. Our observation unveils the microscopic partition dynamics of electrons in co-propagating QH edge states along *p*–*n* junctions.

## Results

### Experimental set-up

Using the conventional exfoliation method^18^, we fabricated a monolayer graphene device. A back-gate electrode is used to tune the carrier density of the whole area, whereas a top-gate electrode can locally tune the density, so that a *p*–*n* junction is formed there^9^,^19^. The insulating layer for the top gate is a crosslinked polymethyl methacrylate (PMMA)^17^,^19^. The optical picture of the device is shown in the upper panel of Fig. 2a, while the lower panel of Fig. 2a represents the one before deposition of the metallic electrodes on graphene. As seen from the picture, the length of the junction is 9.6 μm. We measured the two-terminal differential resistance d*V*_sd_/d*I* of the device at 1.6 K in the QH edge states as well as in the zero magnetic field. Here *V*_sd_ is the source-drain bias voltage across the device and *I* is the current flowing through it. The shot-noise measurements were performed using the experimental set-up schematically shown in Fig. 2b. The current noise power spectral density (*S*_*I*_) was deduced at each source-drain voltage (*V*_sd_) in the QH edge transport regime and in the zero magnetic field.

### Two-terminal resistance measurements

Figure 2c shows the image plot of the differential resistance as a function of the top-gate voltage (*V*_tg_) and the back-gate voltage (*V*_bg_). The cross-sections of the image plot at *V*_bg_=22, 16, 4 and −5 V are shown in Fig. 2d. The image plot shows a cross-like pattern, indicating that the electron transport in graphene can be changed from the unipolar to the bipolar regime by tuning *V*_tg_ and *V*_bg_^9^,^19^, as is also clearly seen in the resistance peaks as a function of *V*_tg_ in Fig. 2d. In Fig. 2c, the horizontal and vertical bright ridges correspond to the charge neutrality, and the bipolar transport regime occurs in regions marked as ‘*pn*' and ‘*np*'. The perfect consistency of our observation with the previous works on the graphene *p*–*n* junctions^9^,^19^ confirms that a single well-defined junction is formed in our device.

By applying a magnetic field of 8 T perpendicularly to the graphene, the device enters the QH regime, and we can measure the QH edge transport through a *p*–*n* junction. Figure 3a represents the image plot of the differential resistance as a function of *V*_tg_ and *V*_bg_, which clearly shows the chequerboard pattern as previously reported^9^. The cross-sections of this image plot at *V*_bg_=22, 16, 4 and −5 V (Fig. 3b) clearly show several resistance plateaus. While the resistances at the plateaus are consistent with the predicted values in half-integer QH effect, 
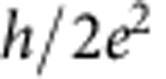
 and 
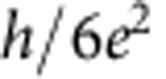
 in the unipolar regime, the resistances at the plateaus are 
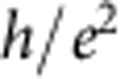
, 
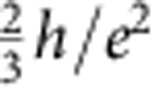
 and 
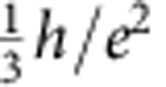
 in the bipolar regime, which are consistent with the previous results^9^,^10^. The quantized values in the bipolar regime are given by 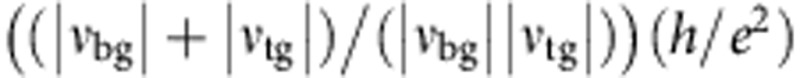, which is obtained on the assumption that the co-propagating QH edge states uniformly mix along the *p*–*n* junction. Here *ν*_tg_ (*ν*_bg_) represents a filling factor of the region beneath the top-gate electrode (the region not covered by the top-gate electrode) (*ν*_tg_, *ν*_bg_=±2, ±6, ...). The unexpected feature around the crossing of the charge-neutrality lines is most probably due to puddles of electrons and holes^9^.

### Shot-noise measurements in QH regime

**Figure 1:**
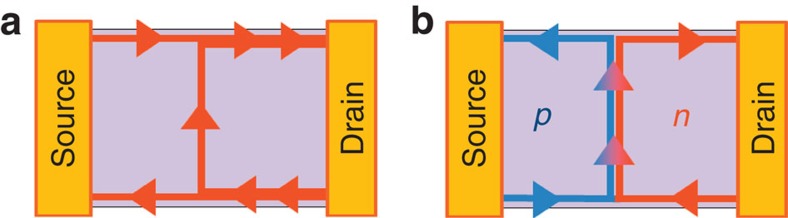
Edge states in the unipolar and bipolar regimes. (**a**) Behaviour of the QH edge states at a junction is schematically shown for the conventional 2DEG case or for the unipolar graphene case. The edge state in the left region and right region circulates in the same direction. (**b**) Behaviour of the QH edge states in the bipolar graphene junction. The edges counter-circulate in the *n* and *p* regions and co-propagate along the junction^10^. The shot noise reveals how the edges mix along the junction.

Now we discuss the results of the shot-noise measurement in this QH regime, which was measured at the centre of each resistance plateau. The red circles and blue circles in Fig. 3c are the typical *S*_*I*_ as a function of the bias voltage *V*_sd_ at (*V*_tg_, *V*_bg_)=(2.5 V, 4 V) and (−0.5 V, 16 V), which correspond to (*ν*_tg_, *ν*_bg_)=(6, −2) and (2, 2), respectively. *S*_*I*_ is defined as the excess noise, where the thermal noise is already subtracted. While the noise at (*ν*_tg_, *ν*_bg_)=(6, −2) (bipolar regime) shows the V-shaped behaviour characteristic of shot noise, the noise at (*ν*_tg_, *ν*_bg_)=(2, 2) (unipolar regime) is strongly suppressed to zero, regardless of *V*_sd_. Thus, there is a fundamental difference in the transport mechanism between the unipolar and bipolar regimes, which cannot clearly be seen by just looking at the source-drain voltage dependence of the differential resistance (Supplementary Fig. 2 and Supplementary Note 2). The absence of the shot noise directly indicates that there is no partition process in the unipolar regime. This reflects the fact that some edge states are connected to both reservoirs, while the others are coupled to only one of the reservoirs as shown in Fig. 1a. In contrast, the finite shot noise in the bipolar regime is a signature of peculiar partition in this regime. This observation is a microscopic evidence of an intermode scattering at the junction (shown in Fig. 1b) as theoretically proposed^10^.

### Fano factors in QH regime

To be more quantitative, we deduce the Fano factor *F* from *S*_*I*_ on each resistance plateau by using the following relation^20^:


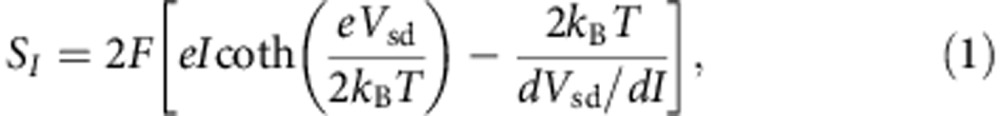


where *k*_B_ and *T* are the Boltzmann constant and the electron temperature of the device, respectively. The Fano factor was extracted with a precision of ±0.01, unless specifically stated otherwise. For example, the numerical fitting to this equation of the data at (*ν*_tg_, *ν*_bg_)=(6, −2) is shown in a solid red curve in Fig. 3c. Clearly, equation (1) reproduces well the experimental result with *F*=0.18. The experimental Fano factors obtained in the same way are compiled in Fig. 4a as a function of *ν*_tg_ and *ν*_bg_. In addition, the experimental results and theoretical predictions of the two-terminal resistance are also compiled in Fig. 4c,d, respectively. The values of *V*_tg_ and *V*_bg_ where we performed the noise measurement are given in Supplementary Fig. 3, Supplementary Table I and Supplementary Note 4. In the unipolar regime, the obtained Fano factors are *F*=0.00, 0.00, 0.02 and 0.07 at (*ν*_tg_,*ν*_bg_)=(2, 2), (−2, −2), (2, 6) and (−2, −6), respectively. Except the (−2, −6) case, which is also well below 0.1, the observed values are very close to zero. This absence of shot noise directly proves that the transport at the junction is dissipation-less in the unipolar regime^10^. This result also guarantees that the shot noise between the graphene and the contacts, if any, would have no influence on the shot noise in the QH regime. On the other hand, the situation is essentially different in the bipolar regime since *F* is clearly enhanced, for example, *F*=0.19, 0.15, 0.18 and 0.18 for (*ν*_tg_, *ν*_bg_)=(2, −2), (−2, 2), (2, −6) and (6, −2), respectively. Although they are finite, the obtained *F*'s are smaller than those expected for a usual quantum point contact with the same conductance^20^, which suggests that a partition peculiar to this case occurs (Supplementary Note 3). We note that the Fano factor is exceptionally as large as 0.42 for (*ν*_tg_, *ν*_bg_)=(−2, 6). At this condition, the resistance does not reach the expected value of 
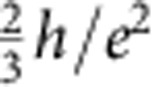
 as seen in the curve of *V*_bg_=22 V in Fig. 3b and also in Fig. 4c. This may be due to the existence of the local regions where bulk of the QH state is not insulating enough.

### Shot-noise measurements and Fano factors at 0 T

As a control experiment, the shot noise is also measured at 0 T. The red circles and blue circles in Fig. 3d are the obtained *S*_*I*_ as a function of *V*_sd_ at (*V*_tg_, *V*_bg_)=(2.5 V, 4 V) and (−0.5 V, 16 V), respectively; the same positions as we obtained for *S*_*I*_ in Fig. 3c. The two curves in Fig. 3d behave almost similarly as a function of *V*_sd_. In other words, the shot noise is finite regardless of the junction nature (‘*pn*' or ‘*nn*') at 0 T, which is significantly different from the results in the QH regime. By using the numerical fitting for the data shown in Fig. 3d, we obtained *F*=0.52 and 0.53 for (*V*_tg_, *V*_bg_)=(2.5 V, 4 V) and (−0.5 V, 16 V), respectively. Figure 4e shows several Fano factors measured at 0 T at the same *V*_tg_ and *V*_bg_ as we measured in the QH regime. It is found that the Fano factors at 0 T are ∼0.5 far from the charge neutral point and they are enhanced around the charge neutral point. Among several experimental works on this topic^21^,^22^,^23^, our result agrees with that for graphene nanoribbon^23^, which reported *F* ∼0.4. According to theoretical studies^24^,^25^, the Fano factors at 0 T strongly depend on the disorder strength, which could explain the present results.

## Discussion

Now going back to the QH regime, we compare our experimental results with the theoretical prediction^10^. The theory argued that, when the electron energy does not relax along the junction and the edge mixing process is incoherent, the shot-noise mechanism is analogous to that in a chaotic cavity, and therefore, the Fano factor in the bipolar regime is given by


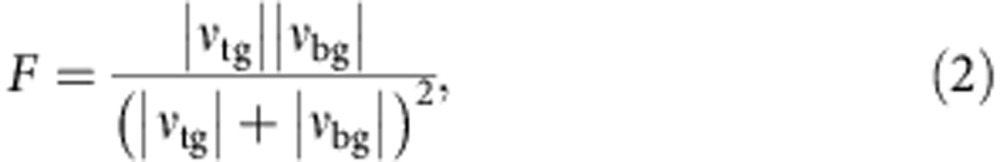


while it should be zero in the unipolar regime. The theoretically predicted Fano factors are compiled in Fig. 4b. For example, the Fano factor in (*ν*_tg_, *ν*_bg_)=(6, −2) and (2, −6) region is 3/16=0.1875, which perfectly reproduces the experimental observation. This consistency could be a microscopic proof that the electron partition takes place on the whole region along *p*–*n* junction^9^,^10^. The QH edges mix to cause equilibration incoherently along the junction.

On the other hand, the experimental results for (*ν*_tg_, *ν*_bg_)=(±2, ±2) and (−6, 2) are smaller than the theoretical results. There are several possible reasons that the experimental value deviates from the theoretical one. For example, it was discussed that the quantum coherence effect and the presence of the energy relaxation may affect the Fano factor^10^. However, in both cases, the Fano factor is expected to be slightly enhanced, and therefore our observation of the reduced values cannot be explained at least by these possibilities. We also note that due to the symmetry reason the same Fano factors are expected between (*ν*_tg_, *ν*_bg_) and (−*ν*_tg_, −*ν*_bg_) and for the exchange of *ν*_tg_ and *ν*_bg_ as seen in Fig. 4b. According to Fig. 4a, this is not always the case; for example, *F*=0.18 and 0.12 are obtained for (*ν*_tg_, *ν*_bg_)=(6, −2) and (−6, 2), respectively. This may indicate that the Fano factor is also sensitive to the detailed geometry of the junction along which the edge mixing occurs.

Finally, we discuss the role of the device mobility. When the graphene device has high mobility, the Zeeman splitting and/or the valley splitting of the Landau levels should be considered^26^, and therefore, it is no more obvious that the present theory^10^ is still applicable. Regarding this point, our device has a low mobility similar to those in the previous works^9^,^11^ so that the energy fluctuation of the Dirac point is larger than the energy of these splittings at 8 T; therefore, the uniformly mixing picture is expected to hold. In high-mobility devices, such splitting^26^ was recently observed. Shot-noise study for these devices will further clarify the unique mixing dynamics in the *p*–*n* junction in graphene bipolar QH regime.

To conclude, by using our high-precision shot-noise measurement, we have successfully established the microscopic dynamics of the peculiar electron partition on the *p*–*n* junction in graphene that the edge mixing occurs when the graphene device is in the bipolar QH regime. Our achievement not only confirms the theoretical prediction but also paves the road toward further exploring the nonequilibrium edge dynamics in wide classes of materials, such as graphene and topological matters.

## Methods

### Device fabrication and the properties

A highly ordered pyrolytic graphite was cleaved by using Scotch tape and was transferred onto a Si substrate covered with 285-nm-thick SiO_2_. We deduced the thickness of the transferred graphene on the substrate by analysing the contrast in the optical picture. Then, we patterned the source and drain electrodes onto the monolayer graphene using a conventional electron-beam lithography and deposited 5-nm-thick palladium and 30-nm-thick gold. Subsequently, we pasted PMMA on the graphene and patterned by electron-beam lithography to make a crosslinked PMMA region as an insulating layer^17^,^19^. Finally, we fabricated the top-gate electrode on this insulating layer. The top-gate electrode consists of 3-nm-thick chromium and 30-nm-thick gold. The mobility of the fabricated graphene is 3.0 × 10^3^ cm^2^ V^−1^ s^−1^ (1.7 × 10^3^ cm^2^ V^−1^ s^−1^) for the condition where the carriers are electrons (holes). These mobilities are high enough for the QH edge state to appear in the magnetic field of 8 T at 1.6 K. From the measurement in the QH regime, the contact resistance is estimated below 100 Ω.

### Set-up for noise measurement

**Figure 2:**
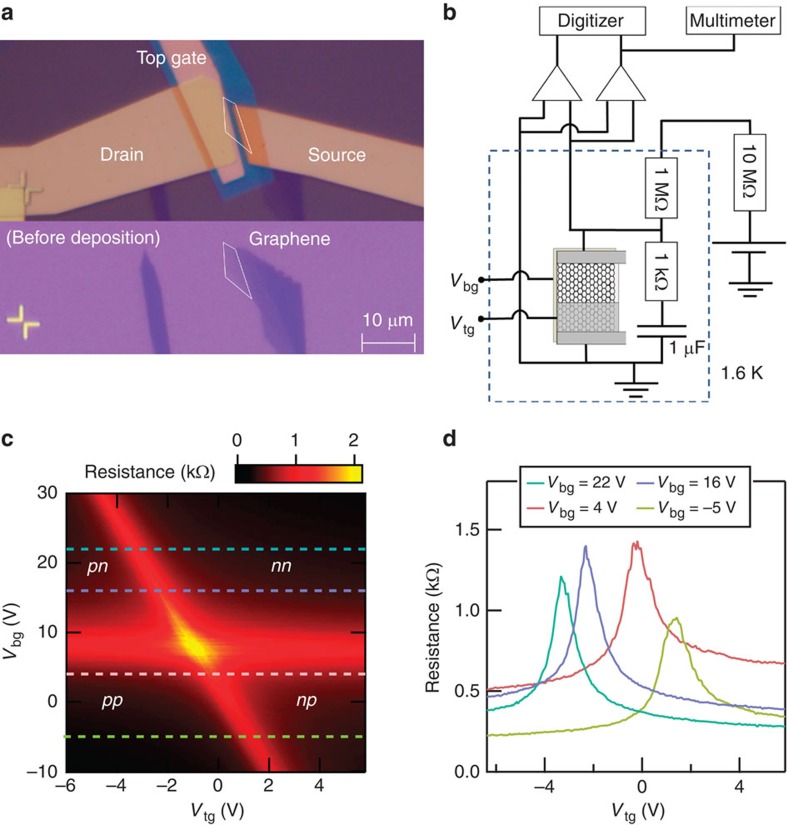
*p*–*n* junction device and its zero-field resistance. (**a**) Upper panel: optical picture of the present graphene device. The source and drain electrodes are attached to the graphene. A top-gate electrode is placed on the insulating layer, PMMA (blue part) covering graphene. Lower panel: the optical picture of the graphene device before the metal deposition. (**b**) Schematic picture of the experimental set-up for the resistance and noise measurement. A resistor of 1 kΩ and a capacitor of 1 μF are inserted in parallel to the graphene device to reduce the impedance. Another resistor of 1 MΩ is placed in series to reduce the external noise. These components are placed in the low-temperature environment at 1.6 K. We measured the voltage noise using a digitizer. (**c**) Image plot of the two-terminal resistance as a function of *V*_tg_ and *V*_bg_ at 0 T. The carrier density of the two regions can be controlled almost independently and the device is tuned to either unipolar or bipolar regime. (**d**) Cross-sections of the image plot of **c** at *V*_bg_=22, 16, 4 and −5 V.

**Figure 3:**
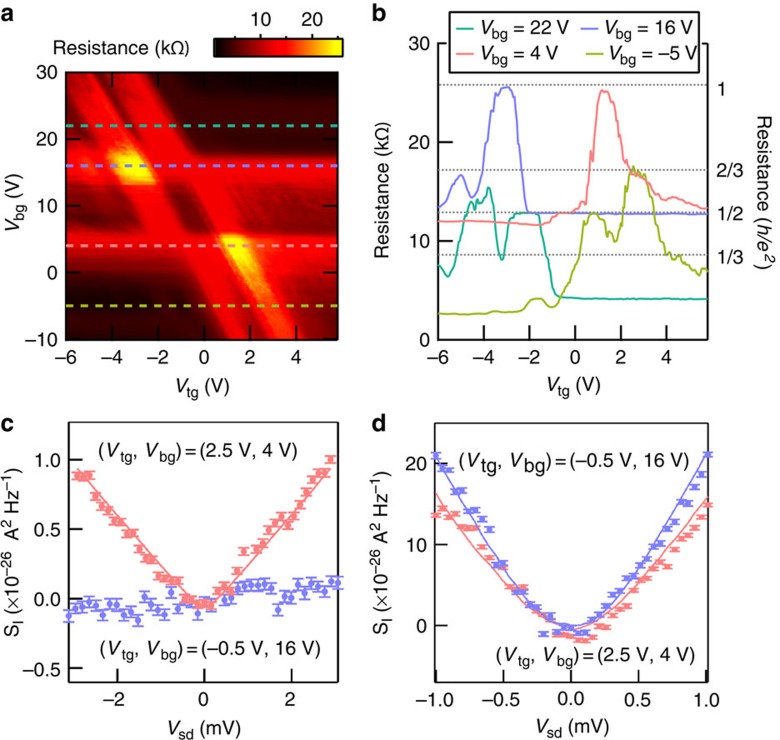
Resistance and shot noise of *p*–*n* junction in the QH regime. (**a**) Image plot of the two-terminal resistance as a function of *V*_tg_ and *V*_bg_ at 8 T. (**b**) Cross-sections of the image plot **a** at *V*_bg_=22, 16, 4 and −5 V. (**c**) *S*_*I*_ as a function of *V*_sd_ at 8 T at (*V*_tg_,*V*_bg_)=(2.5 V, 4 V) and (−0.5 V, 16 V), which correspond to (*ν*_tg_, *ν*_bg_)=(6, −2) and (2, 2), respectively. The solid curve is the result of the numerical fitting to equation (1). (**d**) *S*_*I*_ as a function of *V*_sd_ at 0 T at the corresponding gate voltage conditions for (**c**). The solid curves are the result of the numerical fitting to equation (1).

**Figure 4:**
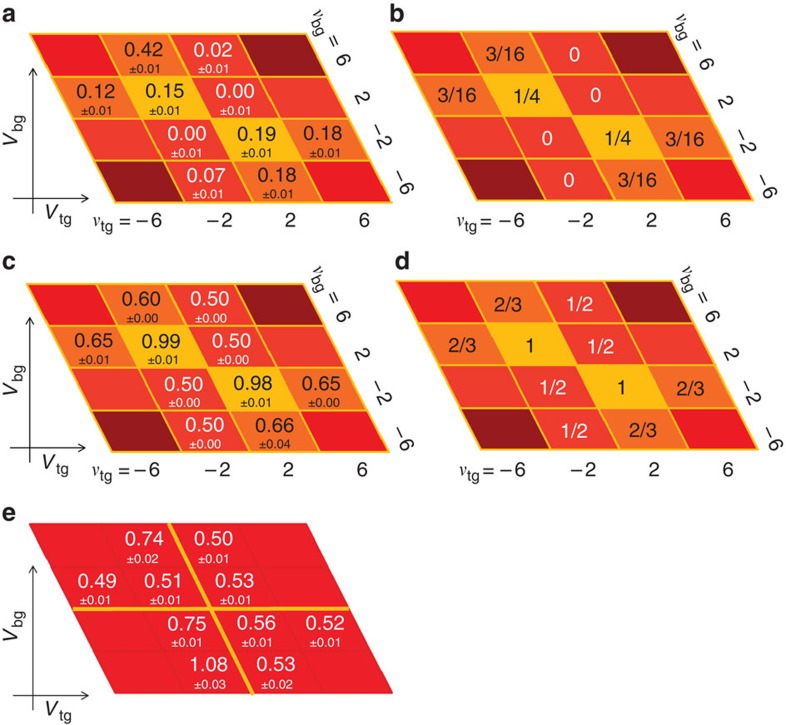
Compilation of experimental and theoretical Fano factors. (**a**) Table of the experimental results of the Fano factor at each (*ν*_tg_, *ν*_bg_). The Fano factors are close to 0 in the unipolar regime, while they are finite in bipolar regime. (**b**) Table of the theoretically predicted Fano factor^10^. (**c**) Table of the experimental results of the resistance at each (*ν*_tg_, *ν*_bg_) in units of *h*/*e*^2^. The experimental results agree well with the theoretical predictions. (**d**) Table of the theoretically predicted resistance^10^ in units of *h*/*e*^2^. (**e**) Table of the experimental results of the Fano factor at 0 T at the same *V*_tg_ and *V*_tg_ corresponding to (**a**). Finite Fano factors are observed in both unipolar and bipolar regime and larger than those at 8 T.

For shot-noise measurement, we inserted a capacitor of 1 μF and a resistor of 1 kΩ in parallel with the device to reduce the circuit impedance, as schematically shown in Fig. 2b. Additionally, a resistor of 1 MΩ was connected in series to reduce the noise from the measurement components at room temperature^27^. In order to reduce the extrinsic noise, the voltages measured by two amplifiers were cross-correlated to yield the voltage noise power spectral density *S*_*V*_ through a fast Fourier transformation^27^,^28^. At a given *V*_sd_, *S*_*V*_ was measured typically between 150 and 180 kHz, where the spectra were frequency-independent. The current noise power spectral density (*S*_*I*_) was deduced at each source-drain voltage (*V*_sd_) (Supplementary Fig. 1 and Supplementary Note 1). The error was also calculated following the previous report^27^.

## Additional information

**How to cite this article:** Matsuo, S. *et al*. Edge mixing dynamics in graphene *p*–*n* junctions in the quantum Hall regime. *Nat. Commun.* 6:8066 doi: 10.1038/ncomms9066 (2015).

## Supplementary Material

Supplementary Information Supplementary Figures 1-3, Supplementary Table 1, Supplementary Notes 1-4 and Supplementary References
