# Supplementary material for: Edge mixing dynamics in graphene p–n junctions in the quantum Hall regime
Source: Nat Commun. 2015 Sep 4;6:8066. doi: 10.1038/ncomms9066 (PMC4569692; doi:10.1038/ncomms9066)
Supplement: Supplementary Information — Supplementary Figures 1-3, Supplementary Table 1, Supplementary Notes 1-4 and Supplementary References [file ncomms9066-s1.pdf]

Supplementary Figures

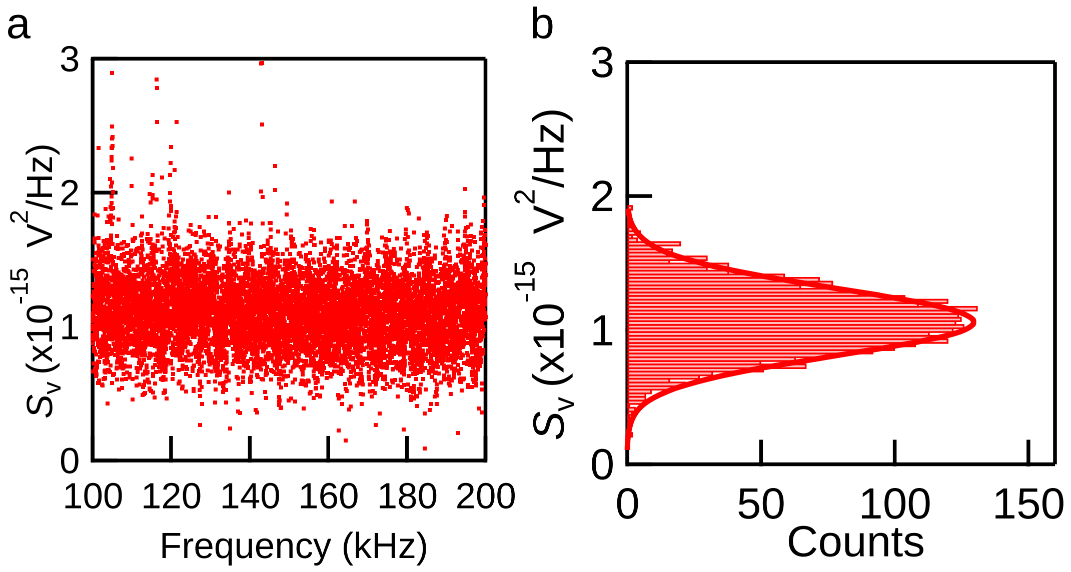

Supplementary Figure 1. **Example of the observed noise spectrum and analysis**: **a,** Typical noise spectral density obtained at $V_{\rm sd} = 0$ mV for $(V_{\rm tg}, V_{\rm bg}) = (2.5\ \mathrm{V}, 4\ \mathrm{V})$ at 8 T. **b,** Corresponding histogram of the voltage noise power spectral density $S_{\rm V}$ between 150 kHz and 180 kHz shown as the red bars. We deduced the spectral density and the error bars by using the numerical fitting of the histogram to the Gaussian as shown by the solid curve.

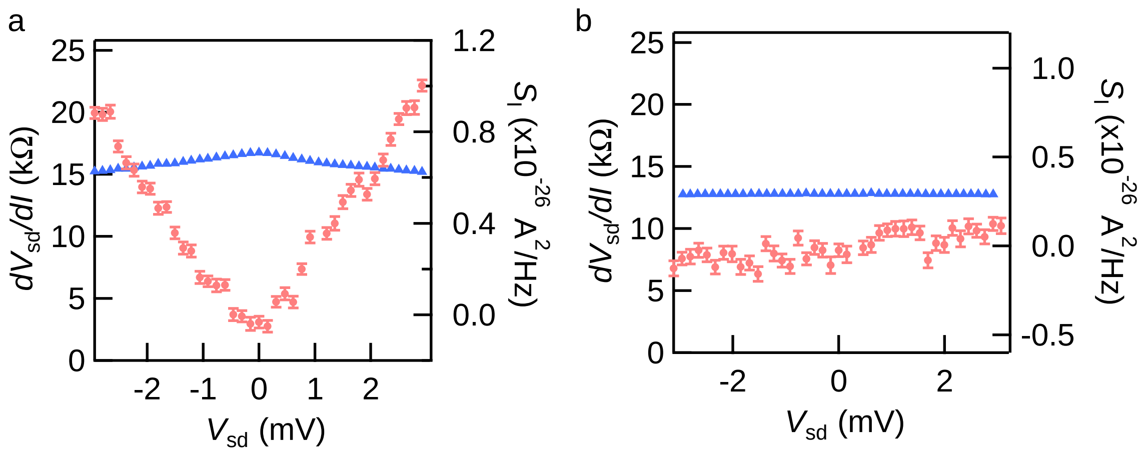

Supplementary Figure 2. **Excess Noise and Differential Conductance**: **a,** S_I and dV_sd/dI as a function of V_sd at $(V_\mathrm{tg}, V_\mathrm{bg}) = (2.5\ \mathrm{V}, 4\ \mathrm{V})$. The red circles and blue triangles represent S_I and dV_sd/dI, respectively. **b,** S_I and dV_sd/dI as a function of V_sd at $(V_\mathrm{tg}, V_\mathrm{bg}) = (-0.5\ \mathrm{V}, 16\ \mathrm{V})$. The red circles and blue triangles represent S_I and dV_sd/dI, respectively.

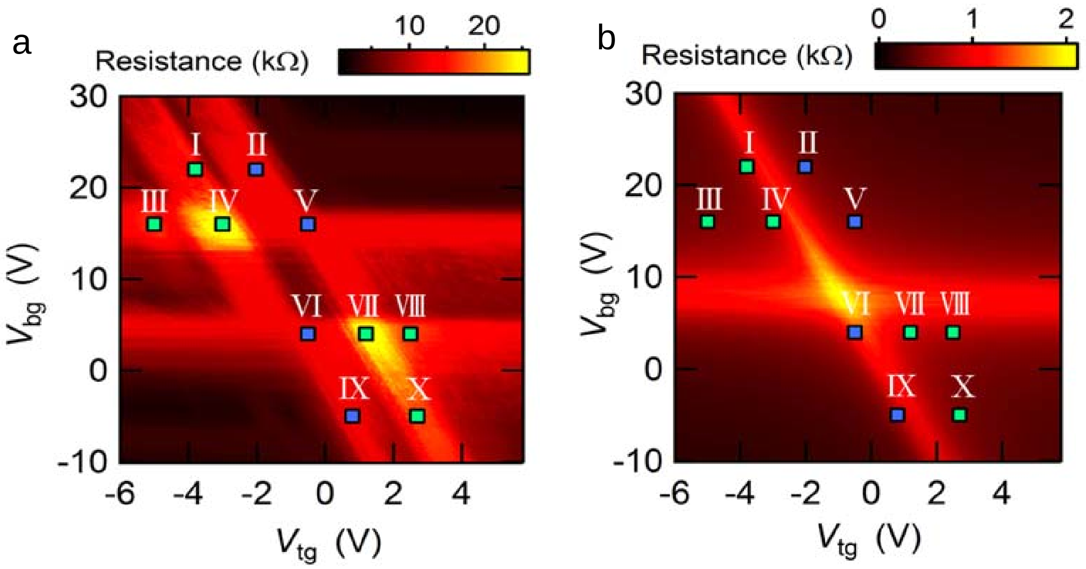

Supplementary Figure 3. **Gate Voltage Positions for the Noise Measurements**: **a,** Resistance as a function of V_{tg} and V_{bg} measured at 8 T. The points indicate the positions we executed the noise measurements. The blue and green points corresponds to the unipolar regime and the bipolar one, respectively. These points are located at the resistance plateaus and are named as I, II, $\cdots$, and X. **b,** Resistance as a function of V_{tg} and V_{bg} measured at 0 T. As in (A), the points indicate the measurement positions. We carried out the noise experiments at 0 T at the same position as we did at 8 T.

Supplementary Table

Supplementary Table I. V_{tg} and V_{bg} of the positions we executed the noise measurements. The names of the positions correspond to those in Supplementary Figures 3a and 3b.

Position	V_{tg} (V)	V_{bg} (V)	unipolar or bipolar
I	-3.8	22	bipolar
II	-2.0	22	unipolar
III	-5.0	16	bipolar
IV	-3.0	16	bipolar
V	-0.5	16	unipolar
VI	-0.5	4	unipolar
VIII	1.2	4	bipolar
VIII	2.5	4	bipolar
IX	0.8	-5	unipolar
X	2.7	-5	bipolar

Supplementary Note 1 : Noise measurement

We measured the voltage noise by the cross-correlation technique [2–5] using the experimental setup as shown in Fig. 2b in the main text. As shown there, we inserted a capacitor of 1 μF and a resistor of 1 kΩ in parallel with the graphene device [6]. If they were not inserted, the voltage signal above $\sim$ 10 kHz would be damped due to the capacitive component of the measurement cables between the device and the room-temperature apparatus, as the device resistance is more than a few kΩ. Moreover, the $1/f$ noise would smear the shot noise contribution in the frequency region lower than a few kHz. By inserting the capacitor and the resistor in this way, we have reduced the circuit impedance to measure the voltage signal above 100 kHz.

We obtained the voltage noise power spectral density (S_V) at each source-drain voltage (V_sd) as follows [3, 4]. Supplementary Figure 1a shows a typical noise spectrum obtained at $V_\mathrm{sd} = 0$ mV for $(V_\mathrm{tg}, V_\mathrm{bg}) = (2.5\ \mathrm{V}, 4\ \mathrm{V})$ at 8 T. The spectrum is almost independent of frequency, as is expected for the thermal noise. The corresponding histogram of the observed S_V between 150 kHz and 180 kHz is shown in Supplementary Figure 1b by the red bar. We performed the Gaussian fitting to this histogram as shown by the solid curve, which enables us to obtain the voltage noise spectral density and its error bar with high accuracy. Finally, we convert the obtained voltage noise power spectral density to the current noise power spectral density (S_I) by using the corresponding differential resistance dV_sd/dI. To further increase the accuracy, we repeated the measurement several times at the same condition and deduced the noise density as an average of them.

Supplementary Note 2 : Differential resistance and shot noise

In Fig. 3c in the main text, we compare S_I as a function of V_sd at $(V_\mathrm{tg}, V_\mathrm{bg}) = (2.5\ \mathrm{V}, 4\ \mathrm{V})$ with that at $(V_\mathrm{tg}, V_\mathrm{bg}) = (-0.5\ \mathrm{V}, 16\ \mathrm{V})$. In Supplementary Figures 2a and 2b, we show the corresponding differential resistance obtained at $(V_\mathrm{tg}, V_\mathrm{bg}) = (2.5\ \mathrm{V}, 4\ \mathrm{V})$ and $(-0.5\ \mathrm{V}, 16\ \mathrm{V})$ as a function of V_sd, respectively. In each figure, the red circles and blue triangles represent S_I and the differential resistance dV_sd/dI, respectively. Although the differential resistance in both figures are almost flat as a function of V_sd, the shot noise behaves very differently between the two. This clearly tells that, from the differential resistance, it is impossible to

distinguish whether or not the electron partition process is present along the junction.

Supplementary Note 3 : Comparison between p-n junction and quantum point contact

Generally, the dimension of the system where electron partition takes place does not necessarily correspond to the one defined by the device geometry, as potential landscapes or defects may create unintended local electron paths as was recently demonstrated [7]. Therefore, we should carefully investigate whether or not the finite Fano factor obtained in the present experiment is intrinsic to the bipolar edge transport. However, the following argument can exclude the above extrinsic possibility. For simplicity, we focus on the Fano factors of 0.19 and 0.15 obtained at $(\nu_{\rm tg}, \nu_{\rm bg}) = (2, -2)$ and $(-2, 2)$, respectively. We consider how different the electron partition along the p-n junction ("one-dimensional contact") is from the conventional quantum point contact (QPC), which might be unintentionally created to cause electron partition in the present junction. By defining T_n as the transmission probability of the n-th channel, the conductance and the Fano factor of QPC are expressed as $\frac{2e^2}{h}\sum_n T_n$ and $\sum_n T_n(1-T_n)/\sum_n T_n$, respectively [8, 9]. We deduce the shot noise in the case that a QPC would be unintentionally created. We safely assume that the spin is degenerated in our experimental condition. The transmission and the number of channels are easily determined by the experimental results of the resistance. Consequently, the Fano factors would be 1/2 at $(\nu_{\rm tg}, \nu_{\rm bg}) = (\pm 2, \mp 2)$, which are much larger than our experimental results. This leads us to conclude that the shot noise observed in our experiments is not originated from an unintentional QPC.

Supplementary Note 4 : Gate voltage conditions for the measurement

In Figs. 4a and 4c in the main text, we show the Fano factors at ten different gate conditions (the top and back gate voltages) at 8 T and at 0 T, respectively. The gate positions are marked in Supplementary Figures 3a and 3b, which are the 2D plot of the resistance at 8 T and 0 T, respectively. These points are chosen such that they are at the resistance plateaus at 8 T. The blue and green points indicate the unipolar regime and bipolar regime, respectively. These points are named as I, II, $\cdots$, and X. The actual top and

back gate voltages for these points are compiled in Supplementary Table I.

Supplementary References

[1] B. Huard, J. A. Sulpizio, N. Stander, K. Todd, B. Yang, and D. Goldhaber-Gordon, "Transport measurements across a tunable potential barrier in graphene," Phys. Rev. Lett. **98**, 236803 (2007).

[2] A. Kumar, L. Saminadayar, D. C. Glattli, Y. Jin, and B. Etienne, "Experimental test of the quantum shot noise reduction theory," Phys. Rev. Lett. **76**, 2778 (1996).

[3] K. Sekiguchi, T. Arakawa, Y. Yamauchi, K. Chida, M. Yamada, H. Takahashi, D. Chiba, K. Kobayashi, and T. Ono, "Observation of full shot noise in cofeb/mgo/cofeb-based magnetic tunneling junctions," Applied Physics Letters **96**, 252504 (2010).

[4] Tomonori Arakawa, Koji Sekiguchi, Shuji Nakamura, Kensaku Chida, Yoshitaka Nishihara, Daichi Chiba, Kensuke Kobayashi, Akio Fukushima, Shinji Yuasa, and Teruo Ono, "Sub-poissonian shot noise in CoFeB/MgO/CoFeB-based magnetic tunneling junctions," Applied Physics Letters **98**, 202103 (2011).

[5] Takahiro Tanaka, Tomonori Arakawa, Kensaku Chida, Yoshitaka Nishihara, Daichi Chiba, KensukeKobayashi, Teruo Ono, Hiroaki Sukegawa, Shinya Kasai, and Seiji Mitani, "Signature of coherent transport in epitaxial spinel-based magnetic tunnel junctions probed by shot noise measurement," Applied Physics Express **5**, 053003 (2012).

[6] Kensaku Chida, Tokuro Hata, Tomonori Arakawa, Sadashige Matsuo, Yoshitaka Nishihara, Takahiro Tanaka, Teruo Ono, and Kensuke Kobayashi, "Avalanche electron bunching in a corbino disk in the quantum hall effect breakdown regime," Phys. Rev. B **89**, 235318 (2014).

[7] Patrick Gallagher, Menyoung Lee, James R. Williams, and David Goldhaber-Gordon, "Gate-tunable superconducting weak link and quantum point contact spectroscopy on a strontium titanate surface," Nature Physics **10**, 748 (2014).

[8] G. B. Lesovik, "Excess quantum noise in 2d ballistic point contacts," Sov. Phys. JETP Lett **49**, 592 (1989).

[9] M. Büttiker, "Scattering theory of thermal and excess noise in open conductors," Phys. Rev. Lett. **65**, 2901 (1990).
